# Donor- and Acceptor-Side Protection Against Photosystem I Photoinhibition in *Arabidopsis thaliana*

**DOI:** 10.3390/ijms27010009

**Published:** 2025-12-19

**Authors:** Marina Kozuleva

**Affiliations:** Institute of Basic Biological Problems of the Russian Academy of Sciences, Federal Research Center “Pushchino Scientific Center for Biological Research of the Russian Academy of Sciences”, 142290 Pushchino, Russia; marina.kozuleva@pbcras.ru; Tel.: +7-4967732488

**Keywords:** photosynthesis, photoinhibition, photosystem I, PGR5, photosynthetic control, fluctuating light

## Abstract

Photosystem I (PSI) photoinhibition (PI(I)) is gaining traction as a potentially more significant threat to plant performance than photoinhibition of photosystem II (PSII). The increased focus is facilitated by the implementation of specific protocols that induce PI(I), such as artificial fluctuating light (FL) and repetitive short saturating pulses (rSPs). rSPs were long considered a specific sub-case of FL. However, recent evidence suggests that PI(I) proceeds via at least two distinct, treatment-dependent mechanisms, leading to damage at the donor or acceptor side of PSI. This discovery suggests that rSPs and FL represent distinct photoinhibitory stresses and that different mechanisms protect PSI against FL and rSPs. This study comparatively analyzed the effects of FL and rSPs on PSI activity in *Arabidopsis thaliana* wild-type plants and a selection of mutants (*pgr5*, *pgrl1*, *stn7*, *tap38/pph1*, and *pgr1*), previously noted or hypothesized to have altered PI(I) sensitivity relative to the wild type. The results of this work, particularly the contrasting sensitivity of *tap38/pph1* compared to the wild type under FL and rSP conditions, strongly suggest that pulsed illumination and fluctuating light are distinct photoinhibitory treatments, and different mechanisms protect PSI against them.

## 1. Introduction

Environmental factors that fluctuate in intensity often induce imbalances in the photosynthetic apparatus, resulting in photoinhibition—a process that affects the reaction centers of both photosystem II (PSII) and photosystem I (PSI). Among these factors, excess light is one of the most ubiquitous and physiologically significant. In the field, the irradiance incident on a single leaf fluctuates constantly due to cloud movement, canopy dynamics, and transient sunflecks, resulting in rapid transitions between low and extremely high light. It is now established that PSI photoinhibition (PI(I)) is more dangerous for plant viability than PSII photoinhibition [1,2,3], as the repair of PSI complexes requires significantly more time than PSII repair, and the consequences are more severe [4,5,6]. In vivo, PI(I) is observed less frequently than PSII photoinhibition because PSI is believed to be better protected against photoinhibitory stresses than PSII [5]. PI(I) was first observed in vivo in 1994 in chilling-sensitive cucumber cultivars under cold stress [7]. Subsequently, various protocols were proposed for targeted PI(I) that minimally affect PSII. Breakthroughs in PI(I) research came with experiments using artificial fluctuating light (FL), which consists of alternating phases of low light and high light (LL and HL, respectively) and is designed to mimic the constantly changing light intensity found in nature [8]. For example, the *Arabidopsis thaliana* mutant lacking a functional Proton Gradient Regulation 5 (PGR5) protein (the mutant line *pgr5-1*), which is deficient in the regulation of Cyclic Electron Transport around PSI (CET(I) [9,10], is unable to grow under FL conditions due to significant PI(I) [11]. Later, a protocol for targeted PI(I) based on a series of repetitive short saturating pulses (rSPs) was introduced [12], which simulates short-duration sunflecks in nature. Both FL and rSPs have been shown to inhibit PSI while leaving PSII largely unaffected [12,13,14].

It is currently established that treatments such as cold stress and HL (which can be constant or alternate with LL, i.e., FL) lead to the destruction of the PSI terminal electron transfer cofactors, the 4Fe-4S clusters F_A_/F_B_, on the acceptor side of the PSI complex [15,16,17,18]. This was particularly evident in *pgr5* under excessive light [16,18]. However, it has recently been demonstrated that rSP treatment does not result in the destruction of the F_A_/F_B_ clusters [17]. Instead, an inconsistency was observed between the kinetics of charge separation in PSI and the signal of the intermediate 4Fe-4S cluster F_X_, which was interpreted as an indication of rSP-induced damage on the donor side of the PSI complex at the site between P_700_ and one of the phylloquinone A_1_ molecules [17]. This breakthrough result suggests that PI(I) proceeds via at least two distinct mechanisms, depending on the initiating treatment, that cause primary photodamage on either the PSI acceptor or donor side.

However, protocols involving pulsed illumination, specifically rSPs, have often been regarded as a variant of FL [3,19]. Moreover, many FL protocols necessitate the application of saturating pulses (SPs) to analyze PSI characteristics, such as its quantum yield [13,14,20]. The periodic application of such pulses (e.g., at 30 or 60 s intervals) can constitute pulsed illumination, which contributes to total PI(I) [12]. Furthermore, rSP protocols are sometimes applied over a strong-light background [21,22], which itself could lead to a loss of PSI activity due to acceptor-side damage.

In this work, we tested the hypothesis that FL and rSP treatments have differential effects on PSI and that distinct protective mechanisms prevent PI(I) under these two stresses. To this end, the effects of FL and rSPs on PSI activity were compared in *Arabidopsis thaliana* wild-type (WT) plants and a selection of mutants previously noted or hypothesized to have altered PI(I) sensitivity relative to the WT. Collectively, the results of this study demonstrate that different protective mechanisms are activated under FL vs. rSP treatments. They indicate that mechanisms regulating electron outflow from the PSI acceptor side are predominantly crucial during treatments that lead to acceptor-side photodamage, whereas mechanisms regulating electron inflow to the PSI donor side are mainly important during treatments that result in donor-side photodamage.

## 2. Results

### 2.1. Comparison of FL and rSP Effects on PI(I) in Arabidopsis WT and Mutants Lacking a Functional PGR5 Protein

**Figure 1:**
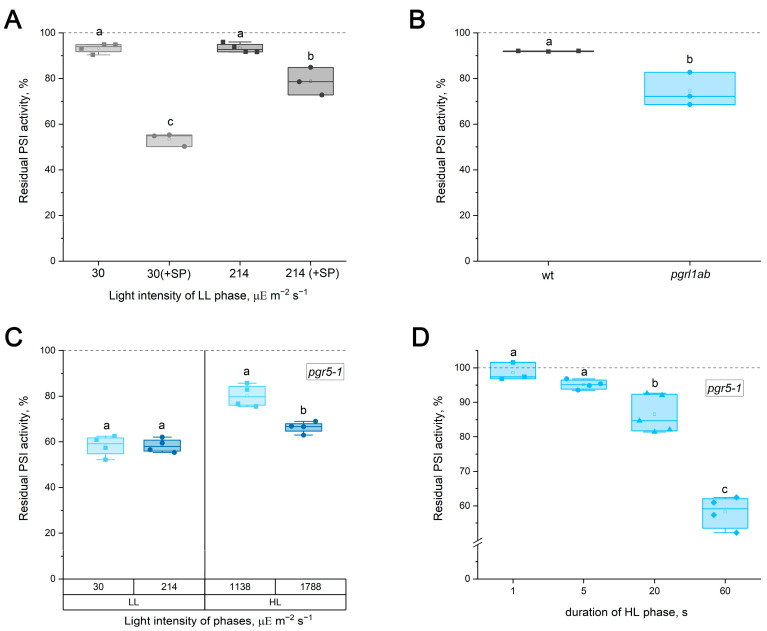
PSI photoinhibition (PI(I) induced by fluctuating light (FL) in *Arabidopsis thaliana* leaves of WT (gray) and PGR5-deficient mutants (blue). (**A**) Effect of routine saturating pulse (SP) application (every 30 s) and low-light (LL) phase intensity on PSI activity in WT. (**B**) FL-induced PI(I) in WT and *pgrl1ab*. (**C**) Effect of light intensity of LL phase (at high-light (HL) phase intensity 1788 µΕ m^−2^ s^−1^) and HL phase (at LL phase intensity of 30 µΕ m^−2^ s^−1^) on FL-induced PI(I) in *pgr5-1*. (**D**) Dependence of FL-induced PI(I) on the HL phase duration in *pgr5-1*. Data represent individual values for 3–5 leaves per variant; boxplots show the median, mean, and interquartile range. Statistical significance was determined by ANOVA. Different letters indicate significant differences (*p* < 0.05).

The initial analysis investigated how the routine application of SPs influences FL-induced PI(I) in WT *Arabidopsis* leaves. FL protocols comprised seven cycles (5 min of 30 µΕ m^−2^ s^−1^ or 214 µΕ m^−2^ s^−1^ and 1 min of 1788 µΕ m^−2^ s^−1^) without SPs and with periodic application of SPs (800 ms of 10,286 µΕ m^−2^ s^−1^) every 30 s under actinic light. Indeed, periodic SP application significantly enhanced PI(I) relative to FL alone (Figure 1A). The loss of PSI activity in the WT without SPs was minor, ranging from 5% to 10% across different plant batches. While in the absence of SPs, the LL phase intensity had no effect on FL-induced PI(I); the inclusion of SPs not only caused greater PSI activity loss but also introduced a dependence on the light intensity of the LL phase (Figure 1A). Therefore, the SP application has additional effects on PI(I). The FL protocol in the experiments described below was utilized without SPs during actinic illumination (unless specified otherwise).

The high sensitivity of PSI to FL-induced PI(I) is well documented in *Arabidopsis* mutants lacking a functional PGR5 protein (PGR5-deficient mutants) [13,14], including when grown under FL conditions that likely did not involve SP application [11,23]. This work utilized the widely studied *pgr5-1* line [9], which has been shown to contain side mutations, some affecting PSI and potentially enhancing the phenotype [23,24]. To complement *pgr5-1*, the *pgrl1ab* mutant line lacking PGR5-like 1 protein (PGRL1), which stabilizes PGR5 in WT [25,26], was also utilized in this study. Knocking out the *Pgrl1* gene prevents PGR5 protein accumulation [26], resulting in a phenotype similar to *pgr5-1*. PI(I) has been minimally studied in *pgrl1ab* thus far.

When subjected to FL without SPs, both *pgrl1ab* and *pgr5-1* exhibited markedly stronger PSI activity loss than the WT (Figure 1). Given the minor PI(I) in the WT, the lack of influence of LL phase intensity on PI(I) was also confirmed for PGR5-deficient mutants (Figure 1C, using *pgr5-1* as an example). In contrast, the HL phase strongly influenced PI(I): increased HL intensity caused greater PSI activity loss (Figure 1C), and extending the HL (at 1788 µΕ m^−2^ s^−1^) duration significantly intensified PI(I), with notable differences at HL durations exceeding 5 s (Figure 1D, Supplementary Figure S1A). These results demonstrate that PSI photodamage occurs during the HL phase.

**Figure 2:**
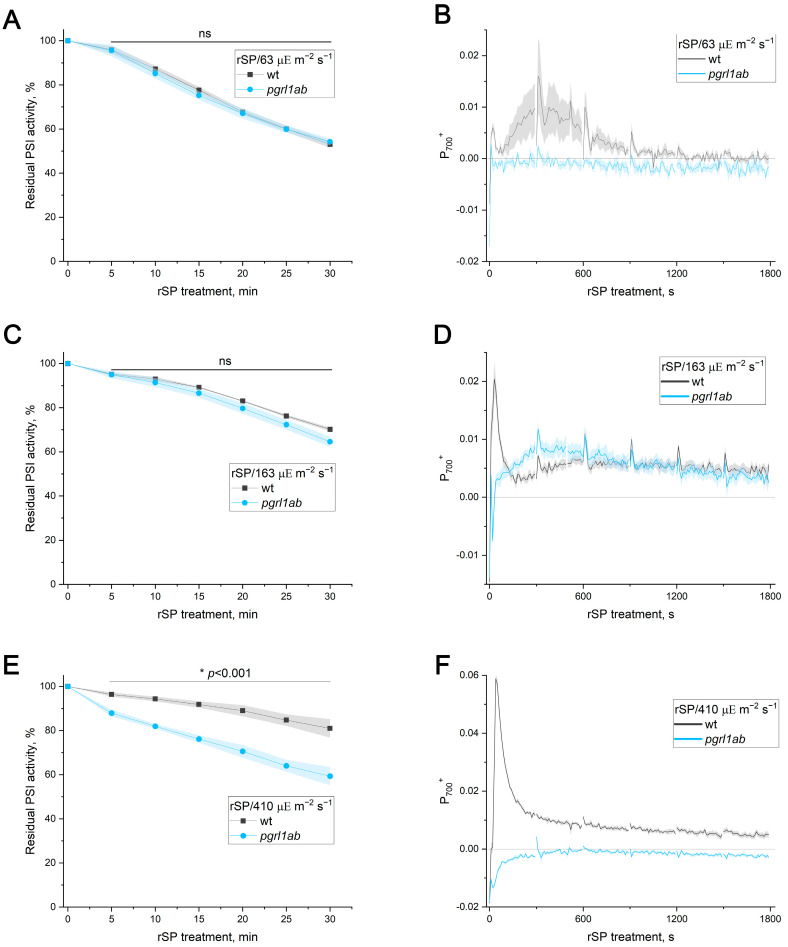
PSI photoinhibition induced by repetitive short pulses (rSPs) and P_700_ redox state in *Arabidopsis thaliana* leaves of WT (gray) and *pgrl1ab* (blue). (**A**,**C**,**E**) PSI activity during rSP treatment applied over background light at 63, 163, and 410 µΕ m^−2^ s^−1^, respectively. (**B**,**D**,**F**) Time courses of oxidized P_700_ signal (P_700_^+^) during rSP treatment at 63, 163, and 410 µΕ m^−2^ s^−1^, respectively. Data represent means of 4–5 individual leaves ± SE. Significant genotype differences at each time point were determined by Two-Sample *t*-Test. Asterisk (*) indicates significant differences with *p* < 0.001, and ns indicates not significant (*p* > 0.05).

Previous work reported comparable rSP-induced PI(I) in WT and PGR5-deficient mutants when pulses were applied in darkness [14,27]. In the present work, rSP treatment was conducted primarily during actinic illumination of varying intensity for a duration of 30 min. Pre-illumination with actinic light alone did not influence subsequent rSP-induced PI(I) (Supplementary Figure S2A). Under rSP treatment over a background of actinic light at 63 and 163 µΕ m^−2^ s^−1^ (rSP/63 and rSP/163), PSI activity loss was similar in the WT and PGR5-deficient mutants (Figure 2A, Supplemental Figure S1B), indicating that the absence of functional PGR5 protein, per se, does not increase susceptibility to rSP-induced PI(I), in contrast to FL-induced PI(I). However, rSP treatment over a 410 µΕ m^−2^ s^−1^ background (rSP/410) resulted in a greater PSI activity loss in *pgrl1ab* compared to the WT (Figure 2E). Importantly, illumination at the same intensity for the same time without rSPs also resulted in significantly greater PI(I) in the mutant than in the WT (residual Pm values were 93.3 ± 0.9% and 89.1 ± 1.2% for the WT and *pgrl1ab*, respectively, *p* < 0.05). PGR5-deficient mutants have previously been noted for a near-complete loss of the oxidized P_700_ signal (P_700_^+^) at light intensities above 200 µΕ m^−2^ s^−1^ [9,26]. The P_700_^+^ signal recorded during rSP treatments is shown in Figure 2B,D,F. Although the P_700_^+^ level in the mutant sometimes differed from the WT under rSP/63 and rSP/163, this did not alter the PSI activity loss kinetics (Figure 2A,C). As expected, the P_700_^+^ signal was absent in *pgrl1ab* during rSP/410 (Figure 2F).

**Figure 3:**
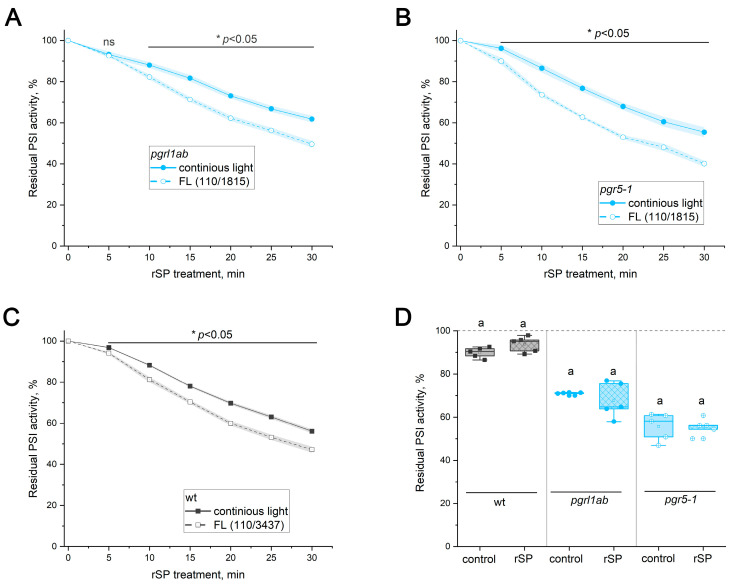
Reciprocal effects of FL and rSP treatments on PI(I) in *Arabidopsis thaliana* leaves of WT (gray squares) and PGR5-deficient mutants (blue circles). (**A**–**C**) Effect of FL pre-treatment on rSP-induced PI(I) in *pgrl1ab* (**A**), *pgr5-1* (**B**), and WT (**C**). For (**A**,**B**), FL protocol included four cycles of 110 µΕ m^−2^ s^−1^ light for 4 min and 1815 µΕ m^−2^ s^−1^ of light for 1 min; for (**C**), FL protocol included six cycles of 110 µΕ m^−2^ s^−1^ of light for of 2.5 min and 3437 µΕ m^−2^ s^−1^ of light for 1 min; continuous 110 µΕ m^−2^ s^−1^ of light for 20 and 21 min. Data represent means of 4–5 individual leaves ± SE. Significant differences between pre-treatment variants for each genotype at each time point were determined by Two-Sample *t*-Test. Asterisks (*) indicate a significant difference (*p* < 0.05), and ns indicates not significant (*p* > 0.05). (**D**) Effect of rSP pre-treatment on FL-induced PI(I) in WT, *pgrl1ab*, and *pgr5-1*. In the case of rSP pre-treatment, Pm value measured after rSP and before FL was taken as 100%. Data represent individual values for 5–6 leaves per variant; boxplots show the median, mean, and interquartile range. Significant differences were determined by ANOVA analysis. Different letters indicate significant differences (*p* < 0.05).

The enhanced PI(I) observed in *pgrl1ab* under rSP/410 may be attributed to progressive acceptor-side PI(I) caused by HL background. To test this, rSP/110 treatment was applied to the WT, *pgrl1ab*, and *pgr5-1* leaves following either (i) pre-illumination at 110 µΕ m^−2^ s^−1^ (which alone caused no PI(I)) or (ii) pre-treatment with four FL cycles (110/1815 µΕ m^−2^ s^−1^), which caused PSI activity losses of 4% in the WT, 12% in *pgrl1ab*, and 19% in *pgr5-1*. Pre-treatment with FL markedly amplified subsequent rSP-induced PI(I) in both PGR5-deficient mutants (Figure 3A,B) but not in the WT (Supplementary Figure S2). Next, the FL pre-treatment protocol was modified to induce a loss of PSI activity in WT comparable to that observed in the PGR5-deficient mutants. To achieve this, the HL intensity was increased to 3437 µΕ m^−2^ s^−1^, and the number of cycles was raised, resulting in a 12% decrease in PSI activity in the WT. Under these conditions, FL pre-treatment stimulated the subsequent rSP-induced PI(I) in the WT too (Figure 3C).

A reciprocal experiment was also performed to examine whether pre-treatment with rSP/darkness, which caused approximately 50% PSI activity loss in all genotypes, affected subsequent FL-induced PI(I) in the WT and PGR5-deficient mutants. Pulsed illumination did not affect subsequent FL-induced PI(I) in either genotype (Figure 3D), indicating that donor-side photodamage resulting from rSPs does not influence subsequent FL-induced PI(I), in contrast to acceptor-side photodamage, which amplifies rSP-induced PI(I).

In summary, PGR5-deficient mutants (*pgr5-1*; *pgrl1ab*), which show strong susceptibility to FL-induced PSI photodamage, do not exhibit greater sensitivity to rSP-induced PI(I) relative to the WT except under actinic light intensities that trigger acceptor-side photodamage.

### 2.2. Photosynthetic Control as a Protective Mechanism

**Figure 4:**
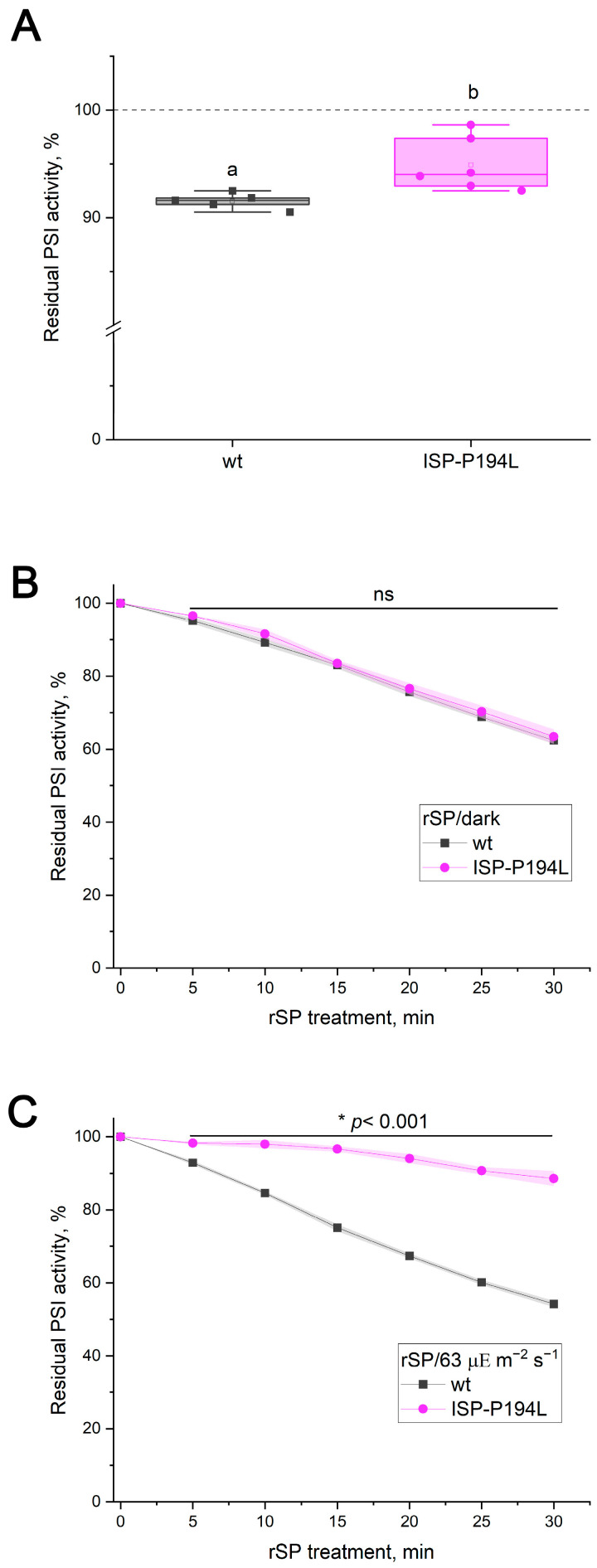
PSI photoinhibition in *Arabidopsis thaliana* leaves of WT (gray squares) and ISP-P194L mutant (purple circles). (**A**) FL-induced PI(I). Data represent individual values for 5–6 leaves per genotype; boxplots show the median, mean, and interquartile range. Significant differences were determined by ANOVA analysis. Different letters indicate significant differences (*p* < 0.05). (**B**,**C**) rSP-induced PI(I) in darkness (**B**) and over background light at 63 µΕ m^−2^ s^−1^ (**C**). Data represent means of 4 leaves ± SE. Significant genotype differences at each time point were determined by Two-Sample *t*-Test. Asterisks (*) indicate a significant difference (*p* < 0.001), and ns indicates not significant (*p* > 0.05).

Photosynthetic control is widely regarded as an important mechanism that mitigates PI(I) [11,14,28,29]. Upon a decrease in thylakoid lumenal pH, plastohydroquinone oxidation in the cytochrome *b*_6_*f* complex slows, thereby limiting the overall photosynthetic electron transport rate [30]. Because this restriction decreases the electron influx to the PSI donor side, photosynthetic control is expected to protect PSI against pulsed illumination. It has also been proposed to protect against FL [11] based on PI(I) observed in the *Arabidopsis* mutant *pgr1* [14] (specifically, ISP-P194L in this work), which carries a point mutation (P194L) in the iron–sulfur protein (ISP) of the cytochrome *b*_6_*f* complex [31]. This substitution shifts the pKa of the proton-accepting H128 residue of the ISP to a more alkaline range [32]. Because deprotonation of H128 is thought to be the rate-limiting step of cytochrome *b*_6_*f* complex turnover upon lumenal acidification [33,34,35], photosynthetic control is activated in the ISP-P194L mutant at more alkaline lumenal pH values than in the WT, meaning lower light intensities. Indeed, ISP-P194L showed statistically significantly lower FL-induced PI(I) than the WT (Figure 4A). Under rSP treatment in darkness, no difference between the WT and the mutant was observed (Figure 4B), indicating that the mutation does not intrinsically alter PSI sensitivity to pulsed PI(I). However, during the rSP/63 treatment, PSI activity was almost entirely preserved in the ISP-P194L mutant, in sharp contrast to the WT (Figure 4C).

### 2.3. STN7 Kinase and TAP38/PPH1 Phosphatase Mutants Under FL and rSPs

**Figure 5:**
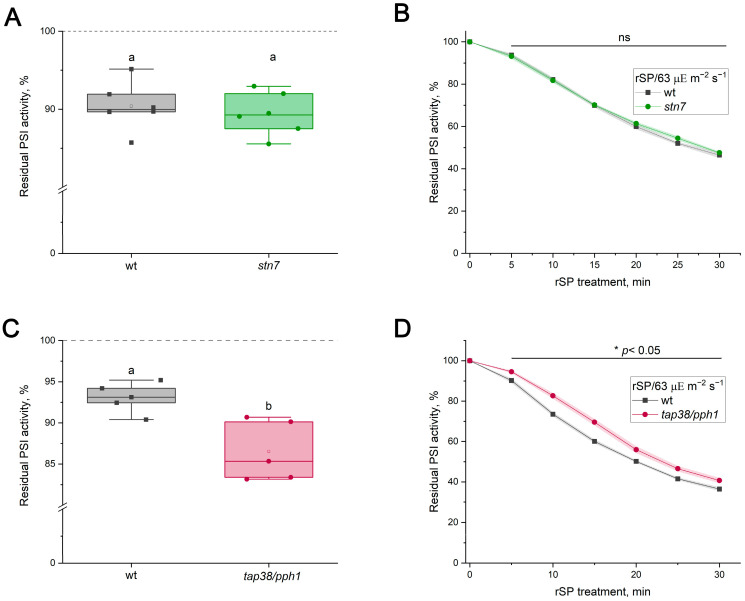
PSI photoinhibition in *Arabidopsis thaliana* leaves of WT (gray squares) and *stn7* (green circles) (**A**,**B**) and WT (gray squares) and *tap38/pph1* (red circles) (**C**,**D**) mutants. (**A**,**C**) FL-induced PI(I). Data represent individual values for 5–6 leaves per genotype; boxplots show the median, mean, and interquartile range. Significant differences were determined by ANOVA analysis. Different letters indicate significant differences (*p* < 0.05). (**B**,**D**) rSP-induced PI(I) over 63 µΕ m^−2^ s^−1^ background light. Data represent the means of 4–6 leaves ± SE. Significant genotype differences at each time point were determined by Two-Sample *t*-Test. Asterisks (*) indicate a significant difference (*p* < 0.05), and ns indicates not significant (*p* > 0.05).

Previous studies reported that Arabidopsis mutants lacking the STN7 kinase (*stn7*) grow more slowly than the WT under FL, which was attributed to enhanced PI(I) in the mutant [8,36]. STN7 is a kinase, which is functionally linked to the cytochrome *b*_6_*f* complex and is responsible for phosphorylating several thylakoid proteins, including light-harvesting complex II (LHCII) [37,38,39,40]. Dephosphorylation of LHCII is catalyzed by a phosphatase TAP38/PPH1 [41,42]. The *tap38/pph1* mutant was reported to exhibit increased PI(I) after exposure to HL for 2 h, in contrast to *stn7* plants, which showed no PSI activity loss [43]. Unexpectedly, in this work, *stn7* showed no detectable differences from the WT under either FL (30/1788 µΕ m^−2^ s^−1^) or rSP/63 treatments (Figure 5A,B). Given that PSI photodamage starts with the first pulses during rSP treatment and this results in a decline in the overall electron transport rate, it is plausible that the developing PI(I) restricts STN7 activation in WT plants, which requires interaction of plastohydroquinone with the cytochrome *b*_6_*f* complex, thereby masking potential differences. To examine this, dark-adapted WT leaves were pre-illuminated for 20 min prior to rSP/63 treatment, a period during which a substantial portion of LHCII is phosphorylated and migrates from PSII to PSI [44]. However, pre-illumination did not provide any effect under rSP/63 (Supplemental Figure S3A). In separate experiments, the influence of rSP treatment on State 2 formation was assessed by measuring the Fr parameter, which reflects changes in chlorophyll *a* fluorescence of leaves induced by state transitions (Supplementary Figure S3B). Indeed, the rSP/63 treatment applied to leaves pre-illuminated for 20 min decreased the Fr parameter, indicating a smaller formation of State 2 at the end of the treatment.

**Figure 6:**
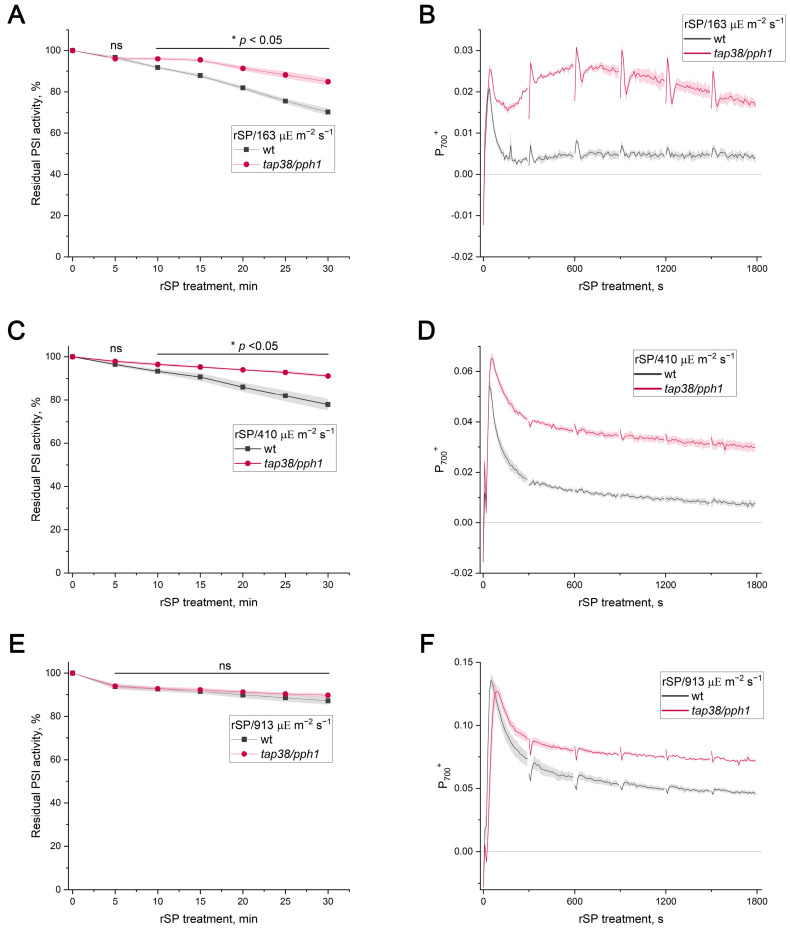
PSI photoinhibition induced by repetitive short pulses and P_700_ redox state in *Arabidopsis thaliana* leaves of WT (gray) and *tap38/pph1* (red). (**A**,**C**,**E**) PSI activity during rSP treatment applied over background light at 163, 410, and 913 µΕ m^−2^ s^−1^, respectively. (**B**,**D**,**F**) Time courses of oxidized P_700_ signal (P_700_^+^) during rSP treatment applied over background light at 163, 410, and 913 µΕ m^−2^ s^−1^, respectively. Data represent means of 4–5 individual leaves ± SE. Significant genotype differences at each time point were determined by Two-Sample *t*-Test. Asterisk (*) indicates significant differences with *p* < 0.05, and ns indicates not significant (*p* > 0.05).

In contrast, *tap38/pph1* exhibited significantly greater loss of PSI activity under FL treatment than WT (Figure 5C). Notably, when FL treatment was performed with periodic SP application, the difference between WT and *tap38/pph1* disappeared (Supplemental Figure S4). During rSP treatments over 63, 163, and 410 µΕ m^−2^ s^−1^ of background light, PSI activity loss in *tap38/pph1* was substantially lower than in the WT (Figure 5D and Figure 6A,C). However, no difference was detected under rSP/913 (Figure 6E). The enhanced PSI stability in *tap38/pph1* against rSP was accompanied by a higher level of the P_700_^+^ signal relative to the WT (Figure 6B,D). Intriguingly, even under the rSP/913 treatment, where PI(I) levels were similar in the WT and *tap38/pph1*, the mutant still exhibited a higher steady-state P_700_^+^ level (Figure 6F), indicating that increased P_700_^+^ does not necessarily translate into greater PSI protection against pulse light.

**Figure 7:**
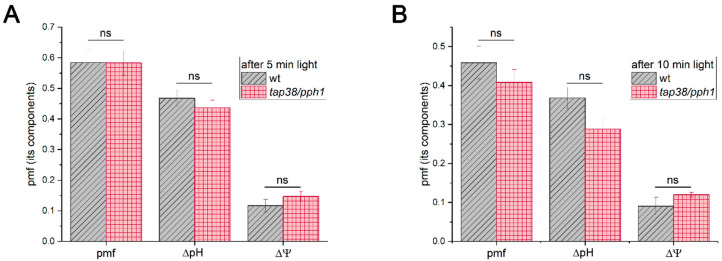
Effect of illumination duration on pmf and its components (ΔpH and ΔΨ) in WT and *tap38/pph1*. Leaves were illuminated at 163 µΕ m^−2^ s^−1^ for 5 min (**A**) or 10 min (**B**). Data represent means of 6 leaves ± SE. Genotype differences were determined by Two-Sample *t*-Test; ns indicates not significant (*p* > 0.05).

The enhanced PSI stability in *tap38/pph1* under rSPs could potentially be explained by a more effective operation of photosynthetic control, for instance, due to larger ΔpH values in the mutant. Although earlier studies reported no difference in pmf between the WT and *tap38/pph1* in low and moderate light [43], genotype-specific variation might emerge under the growth and measurement conditions used here. However, measurements of total pmf and its components after 5 and 10 min at 163 µΕ m^−2^ s^−1^ revealed no difference between the WT and *tap38/pph1* (Figure 7). These results argue against photosynthetic control as a reason for enhanced PSI stability under pulsed illumination in *tap38/pph1*.

Taken together, all results, and particularly the opposing responses of *tap38/pph1* to FL versus rSPs, demonstrate that the mechanisms protecting PSI under FL and pulsed illumination are fundamentally distinct.

## 3. Discussion

### 3.1. Mechanistic Distinction Between FL and rSPs

The present work directly compared two commonly used approaches for inducing PI(I), FL and rSPs, to determine whether the mechanisms protecting PSI under these stresses are distinct. It has recently become clear that PI(I) proceeds via at least two mechanisms depending on the photoinhibitory treatment [17]. The findings of [17] raise the critical issue that FL and rSP protocols must be clearly separated to elucidate the mechanisms of PI(I) under various conditions. The results presented here strongly support the conclusion that FL and rSPs do not represent interchangeable versions of the same stress but instead induce fundamentally different forms of PSI photodamage. If rSPs were a merely specific case of FL, the background actinic light during rSPs and pulses would represent the LL and HL phases of the FL protocol, respectively. It was reported earlier that the intensity of the background light is critically important for rSP-induced PI(I) [12]. Using WT plants and PGR5-deficient mutants, for which the hypersensitivity of PSI to FL is well documented and unambiguous [8,14], it was demonstrated that the intensity of the LL phase had no detectable effect on the level of FL-induced PI(I) (Figure 1), whereas the loss of PSI activity was tightly linked to the HL phase. Short HL exposures did not cause detectable FL-induced PI(I) in PGR5-deficient mutants (Figure 1D, Supplementary Figure S1), consistent with the observation that at background intensities of 63 µΕ m^−2^ s^−1^ or 163 µΕ m^−2^ s^−1^, the rSP treatment did not differentiate between the WT and PGR5-deficient mutants (Figure 2A,C; Supplementary Figure S1). Thus, the duration of the HL phase is a critical factor for distinguishing between FL and rSPs. Moreover, if rSPs were a specific case of FL, PGR5-deficient mutants would exhibit greater sensitivity to rSPs than the WT at all background light intensities, which is clearly not the case. Differences emerged only when background light itself was sufficiently strong to induce acceptor-side photodamage in PGR5-deficient mutants even in the absence of pulses (Figure 2E). Indeed, pre-exposure to FL, which damages the acceptor side, enhanced the subsequent rSP-induced PI(I) (Figure 3); conversely, the reverse sequence of exposures had no effect. If FL and rSPs were identical stressors, the order of their application would be irrelevant. Finally, the opposing sensitivity of *tap38/pph1* to FL and rSPs (Figure 5C,D) unequivocally demonstrates that the FL and rSP protocols employed in this work induce distinct PI(I) processes.

Many protocols for targeted PI(I) include HL of 1 s or shorter, and these treatments are often assumed to simulate FL [19,45]. However, the results of this work demonstrate that short HL pulses do not simulate FL, and therefore, the PSI protection mechanisms identified using such pulse-based protocols, as well as the physiological outcomes of these treatments, should be interpreted within the framework of pulsed illumination, not FL.

Artificial FL mimics the continuous changes in irradiance experienced by leaves in open habitats due to shifting cloud cover. In contrast, pulsed illumination mimics sunflecks—brief flashes of sunlight lasting hundreds of milliseconds that reach the understory or leaves in the lower canopy under shade. Leaves of plants growing in open environments hardly experience sunflecks, whereas changes in irradiance caused by cloud movement are far less relevant in the understory compared to leaves exposed to direct sunlight. Thus, it is reasonable to assume that plants may have evolved different protective strategies to cope with short (milliseconds) versus prolonged (tens of seconds) fluctuations in light.

### 3.2. Warning on the Risks Associated with the Application of Saturating Pulses

A fundamental point in comparing the two treatments was the exclusion of SPs from the FL protocol. In [12], it was shown that the degree of PSI damage is proportional to the frequency of high-intensity pulses: the greatest effect was observed with pulses applied every 10 s, although pulses at 30 and 60 s intervals also led to PSI activity loss. The present work showed that the periodic application of SPs (800 ms, 10,286 µΕ m^−2^ s^−1^) further exacerbates the decline in PSI activity during FL (Figure 1A). In [13], it was reported earlier no difference in the degree of FL-induced PI(I) when periodically applying SPs (250 ms duration, 7000 µΕ m^−2^ s^−1^) every 30 s. However, these were not routinely applied SPs: the authors stated, “To oxidize the intersystem electron carriers, far-red light was applied from 200 ms before the start of the SP to its cessation”. This short exposure to far-red light likely initiated the oxidation not only of upstream electron chain components but also of PSI itself. Because P_700_ oxidation is known to protect PSI from pulsed illumination [28], such pulses are intrinsically less harmful than SPs delivered without far-red pre-illumination.

According to the results of this work, routinely applied SPs (i.e., without far-red light) enhance PI(I) investigated under FL (Figure 1A), likely resulting from donor-side damage, whereas FL itself causes acceptor-side damage. Thus, an FL protocol that includes SPs does not allow for the clear elucidation of PSI protection mechanisms on the acceptor side because SPs introduce donor-side damage. This is illustrated by the lack of influence of the LL phase intensity on FL-induced PI(I) in our experiments when SPs are omitted, and its reappearance when SPs are included (Figure 1A), which is in accordance with a previous study that noted that pulse-induced PI(I) is more severe at lower background light intensities [12]. Another example of a mistaken conclusion that can be drawn based on the FL protocol with SPs is the lack of differences between *tap38/pph1* and the WT when processing such a protocol (Supplementary Figure S4).

The results of this work are also relevant beyond PI(I) research. SPs are routinely applied in chlorophyll fluorescence measurements to analyze PSII performance and non-photochemical quenching. Frequent application of SPs during long experiments can induce PI(I), which may remain unnoticed if Pm is not controlled using far-red light. Gradual loss of PSI activity will inevitably affect the entire electron transport chain, ultimately reducing PSII activity as well. This effect will be particularly pronounced at lower light intensities. Such experimental conditions may lead to misinterpretation of results, especially when comparing different light intensities or genotypes with altered regulation of electron flow to PSI (e.g., *tap38/pph1*) or electron outflow from PSI.

### 3.3. Mechanisms Mitigating PSI Photoinhibition

Characterizing specific protective mechanisms of PSI against photoinhibition was not the primary aim of this study; however, the obtained data provide useful insights into mechanisms mitigating PI(I). Because PSI photodamage under both FL and rSPs is triggered by ROS formed within PSI, protective mechanisms must ultimately act by reducing the probability of generating damaging ROS. The nature of photodamage caused by FL and rSPs, therefore, needs to be considered first.

Acceptor-side photodamage of PSI

Under chilling and HL stress, the initial damage occurs at the [4Fe-4S] clusters of PSI [7,16,17,18]. In vitro, exogenous H_2_O_2_ accelerates PSI activity loss in spinach thylakoids [46], whereas hydroxyl radical (HO^•^) scavengers prevent PI(I) [47]. Importantly, methyl viologen, a highly efficient artificial electron acceptor from the F_A_/F_B_ clusters, strongly suppresses PI(I) [46,47] and lowers HO^•^ generation in thylakoids [48]. As methyl viologen itself enhances H_2_O_2_ production in thylakoid suspension during illumination, these findings indicate that the critical factor in F_A_/F_B_ photodamage is not the absolute amount of H_2_O_2_ accumulating near the acceptor side of PSI but rather the inefficiency of electron outflow from PSI. Together, these results support the following scheme of acceptor-side PSI photodamage [49]:

(i) PSI cofactors reduce O_2_ to superoxide radical (O_2_^•−^), which further dismutates to H_2_O_2_; the chloroplast antioxidant system neutralizes H_2_O_2_.

(ii) When electron outflow from PSI is restricted, the reduced terminal clusters F_A_/F_B_ catalyze HO^•^ formation from H_2_O_2_ via a Fenton-type reaction prior to H_2_O_2_ detoxification by antioxidants.

Thus, the mechanism mitigating the acceptor-side PI(I) should primarily decrease HO^•^ formation by promoting efficient oxidation of F_A_/F_B_ clusters.

An earlier concept proposed that a sudden shift in HL causes a rapid increase in electron flow from PSII to PSI, while CO_2_ fixation kinetics remain temporarily slow [50] due to slower stomata opening and the activation of the Calvin–Benson–Bassham (CBB) cycle. Indeed, increasing CO_2_ concentration from 400 to 800 ppm greatly improved PSI stability under FL in *Arabidopsis* [51], a C3 species for which 400 ppm is suboptimal for efficient CBB cycle operation [52]. Since the CBB cycle is the major electron sink for PSI, its optimal function should serve as a primary protective mechanism against FL-induced PI(I). The present work showed that PSI photodamage occurs specifically during the HL phase and requires several seconds of HL exposure (Figure 1C,D, Supplementary Figure S1A), consistent with slower activation of the CBB cycle.

However, linear photosynthetic electron transport cannot provide sufficient ATP for CO_2_ assimilation [53]. Thus, CET(I) is crucial for supporting the CBB cycle in angiosperms by providing additional ATP. Although the precise role of PGR5 remains debated [54,55], impaired regulation of CET(I) in PGR5-deficient mutants under changing environmental conditions is still the most widely accepted hypothesis. Specifically, these mutants are unable to upregulate CET(I) and optimize CO_2_ assimilation when light intensity increases [10,56]. Another characteristic of PGR5-deficient mutants is the near absence of the P_700_^+^ signal under HL illumination [9,26]. Infiltration of *pgr5-1* leaves with methyl viologen restores the P_700_^+^ signal [9], indicating that the absence of the P_700_^+^ signal in untreated *pgr5-1* leaves is primarily caused by impaired electron outflow from PSI. As a result, electrons predominantly return to P_700_^+^ via charge recombination, thereby suppressing the detectable P_700_^+^ signal in the mutants. The present findings strongly support this explanation for the enhanced FL-induced PI(I) in PGR5-deficient plants. Reduced CET(I) activity in HL has also been previously reported in *tap38/pph1* [43], which may also explain its greater sensitivity to FL (Figure 5C). The reasons for the lower CET(I) activity in *tap38/pph1* remain unclear and may be related to its inability to regulate grana size [43], which can influence CET(I) [57].

Photosynthetic control has also been proposed as a protective mechanism against FL [11], although experimental evidence regarding its effect on ROS production in PSI is lacking. It was suggested that slowing electron donation to PSI at the cytochrome *b_6_f* complex, which keeps P_700_ in an oxidized state, can ensure charge recombination from F_A_/F_B_ clusters (i.e., their oxidation), thereby decreasing the probability of their reaction with H_2_O_2_ [35]—a proposition that still requires experimental verification. Indeed, the greater PSI stability against FL in the ISP-P194L mutant (Figure 4A), which activates photosynthetic control at a more alkaline lumenal pH [32], may support this idea. However, the higher pH threshold for photosynthetic control activation in ISP-P194L is likely most relevant in low and moderate light when lumenal pH remains high. Under HL, lumenal pH drops in the WT as well, so the genotypic difference in photosynthetic control activation may diminish. In addition, CET(I) may also be upregulated in the ISP-P194L mutant as a secondary or compensatory response, which could likewise contribute to its enhanced PSI stability under FL-induced PI(I). Therefore, to draw a definitive conclusion about the basis of the mutant’s resistance to acceptor-side photodamage, data on the regulation of CET(I) and the CBB cycle in ISP-P194L under increasing light intensities are needed. Such in vivo data are currently lacking.

State transitions have also been proposed as a protective mechanism against FL based on smaller rosette size and reduced PSI content in *stn7* plants grown under FL [8,36]. Unexpectedly, mature *stn7* plants grown at 100 µΕ m^−2^ s^−1^ showed no difference from the WT in PSI sensitivity under FL (Figure 5A). This contradiction may have several explanations. It is possible that the process catalyzed by the STN7 kinase may be essential for PSI stability during early vegetation. However, see [40], which showed that STN7 kinase regulates processes in the chloroplast during senescence. Another explanation is that STN7 kinase may regulate a signaling pathway whose disruption affects PSI stability only after prolonged FL exposure. Indeed, a signaling role for STN7 kinase has been previously suggested [58,59].

### 3.4. Donor-Side Photodamage of PSI

A detailed analysis of rSP-induced PI(I) in spinach chloroplasts [28] proposed that both O_2_^•−^ produced at the A_1_-sites and singlet oxygen (^1^O_2_) produced via P_700_ triplet (^3^P_700_) can contribute to PSI damage, although the involvement of ^1^O_2_ appears more likely (see [35] for details). Therefore, mechanisms that mitigate donor-side photodamage of PSI should primarily decrease the probability of ^1^O_2_ generation, as well as the probability of O_2_^•−^ generation at the A_1_-sites. This can be achieved by slowing electron donation to PSI, thereby keeping P_700_ in the oxidized state and decreasing PSI turnover during HL pulses to prevent over-reduction in PSI. By contrast to chilling or HL stress, rSPs do not induce F_A_/F_B_ destruction [33]. Thus, it seems plausible that processes ensuring efficient electron outflow from PSI do not contribute to the protection of PSI against rSPs. Indeed, altering CO_2_ partial pressure had no effect when Arabidopsis leaves were treated with HL pulses every 5 s against a 35 µΕ m^−2^ s^−1^ background light [60], in contrast to FL, where increasing CO_2_ concentration mitigated PI(I) in Arabidopsis leaves [51].

Enhanced PSI stability under rSPs applied in darkness was previously reported for an *Arabidopsis* mutant with reduced plastocyanin content due to impaired Cu transport into chloroplasts, in which P_700_⁺ was reduced more slowly [27]. This strongly suggested that photosynthetic control may be a major protective mechanism against rSPs. The present study demonstrates, for the first time, that photosynthetic control indeed protects PSI under rSPs applied under actinic light (Figure 4C). Since no difference between genotypes was observed in the dark (Figure 4B), these results confirm that the protective mechanisms are light-dependent. It was also reported earlier that photosynthetic control is activated at lower irradiance in shade plants than in sun plants [61], supporting the idea that shade-adapted species may upregulate PSI protection mechanisms specifically against sunflecks.

In *tap38/pph1*, PSI exhibits greater stability against pulsed illumination too (Figure 5C and Figure 6). However, the pmf magnitude did not differ between the WT and *tap38/pph1* (Figure 7), indicating that photosynthetic control is unlikely to be enhanced in *tap38/pph1* compared to WT. This explanation cannot be entirely ruled out, e.g., due to as-yet unknown changes in a regulatory process at the cytochrome *b*_6_*f* complex level, which might cause photosynthetic control to be activated at a more alkaline lumenal pH in *tap38/pph1* than in the WT. The lack of LHCII dephosphorylation in *tap38/pph1* impairs grana size regulation, causing a slightly faster P_700_^+^ reduction rate in the mutant than in the WT [43]. This effect, however, should have led to a more sensitive PSI for rSP-induced PI(I) in *tap38/pph1*, not a more stable one, given that the slowdown of electron transport to PSI caused by photosynthetic control in the ISP-P194L mutant has a clear protective effect on PSI stability against rSP-induced PI(I) (Figure 4C). The greater PSI stability against pulsed illumination in *tap38/pph1* relative to WT can be explained by a larger population of PSI complexes in State 2 in the mutant. However, rSP-induced PI(I) in the WT was unaffected by pre-illumination, which stimulated State 2 formation prior to rSP treatment (Supplemental Figure S2); moreover, no difference in rSP-induced PI(I) between the WT and *stn7* was observed (Figure 5B). These results seem to argue against state transitions as an important mechanism for mitigating pulse-induced PI(I). However, the effect of rSPs on electron transport and on the activity of enzymes involved in state transitions must also be taken into account. By inhibiting PSI, rSP treatment reduces electron flow, which in turn lowers the frequency of plastohydroquinone interactions with the cytochrome *b_6_f* complex and may also diminish the activation of the STN7 kinase, whereas TAP38/PPH1 phosphatase is thought to remain constitutively active. As a result, the population of PSI complexes in State 2 may decrease, which was observed in this study as a reduction in the Fr parameter (Supplementary Figure S2B). Alternatively, *tap38/pph1* was previously noted to have a high spillover level, probably via the LHCII-bridge located between PSI and PSII [62]. Recently, an increased capacity for spillover was used to explain the enhanced PI(I) stability of *Alocasia odora* plants grown in LL [63]. Furthermore, the protective effect of far-red light against PI(I) induced by HL pulses [19] may be related to spillover, considering that far-red light is enriched in green shade, which is the natural habitat of shade-tolerant plant species that experience sunflecks. It is plausible that spillover is yet another mechanism mitigating PI(I) under pulsed illumination. Further studies are required to test this hypothesis.

Experiments testing the effect of FL pre-treatment on subsequent rSP-induced PI(I) in the WT and PGR5-deficient mutants demonstrated that a partial inhibition of PSI activity stimulated the subsequent rSP-induced PI(I) (Figure 3). Thus, increasing the population of PSI complexes with damaged F_A_/F_B_ clusters promotes further donor-side photodamage. This may be due to an increased probability of charge recombination from intermediate electron transfer cofactors in the PSI complexes with damaged F_A_/F_B_, leading to a higher chance of ^3^P_700_ [64]. Indeed, a ^3^P_700_ signal was registered in PSI complexes from cucumber leaves treated with rSP [17]. ^3^P_700_ can then lead to the generation of ^1^O_2_, one of the most deleterious ROS, whose involvement in rSP-induced PI(I) has been confirmed in vitro by a protective effect of a ^1^O_2_ quencher [65]. O_2_^•−^, generated by reduced phylloquinone molecules at A_1_-sites [66], has also been proposed to cause rSP-induced PI(I) [17,65]. In accordance with this, the destruction of all 4Fe-4S clusters in PSI, F_X_, F_A_, and F_B_ enhanced O_2_^•−^ formation at A_1_-sites [66]. Therefore, acceptor-side photodamage promotes the development of donor-side photodamage, which has been clearly observed in this work under rSP treatment; however, it should also occur under HL. Under such conditions, protective mechanisms that regulate electron inflow to the donor side of PSI should alleviate secondary donor-side photodamage.

## 4. Methods

### 4.1. Plant Material

*Arabidopsis thaliana* wild-type (WT) plants (lines Col-0 and Col-5) and mutants were grown in a controlled growth chamber under a 10 h light–14 h dark photoperiod at 100 µΕ m^−2^ s^−1^ until plants reached 5–7 weeks of age. Seeds for Col-5, *pgr5-1*, and *pgr1* (ISP-P194L) were kindly provided by Dr. Toshiharu Shikanai (Kyoto University, Kyoto, Japan); seeds for *stn7*, *tap38/pph1*, and *pgrl1ab* were kindly provided by Dr. Dario Leister (Ludwig-Maximilians-Universität München, Planegg-Martinsried, Germany).

### 4.2. Photosystem I Photoinhibitory Treatments

PI(I) was induced in fully mature, dark-adapted leaves attached to the plant. Each leaf was positioned between the DUAL-DB and DUAL-EDP700 modules of the DUAL-PAM 100 system (Heinz Walz GmbH, Effeltrich, Germany), which served both as the illumination source and as the PSI activity measuring system. All remaining leaves on the plant were shielded from light using an opaque blackout barrier covering the instrument modules. For all protocols, the initial maximal P_700_ oxidation level (Pm) of the dark-adapted leaves was determined by applying an SP under far-red light.

The FL treatment followed previously published protocols [14,67]. The standard FL regime consisted of seven cycles, each consisting of 5 min of LL (30 µΕ m^−2^ s^−1^) and 1 min of HL (1788 µΕ m^−2^ s^−1^), except where otherwise specified. After the final cycle, the leaf was kept in darkness for 3 min, and the post-illumination Pm was measured. Residual PSI activity was evaluated as the Pm after treatment relative to the initial Pm (%).

The standard rSP regime consisted of a series of short SPs (300 ms of 13,953 µΕ m^−2^ s^−1^), applied every 10 s for 30 min under background actinic light, the intensity of which varied depending on the experimental objectives. Every 5 min, actinic light and the rSPs were paused, and far-red light was applied to determine transient Pm, analogous to [68]. PSI activity change was assessed as the ratio of transient Pm relative to the initial Pm (%) at each time point. In separate experiments, it was verified that interruptions for transient Pm determination did not influence residual PSI activity after 30 min of rSP.

### 4.3. Photosystem I Activity Measurement

PSI activity in leaves was monitored by recording redox changes in P_700_ via absorbance differences at 830 and 875 nm using DUAL-PAM 100. Pm was determined by applying an SP under far-red light. In the rSP treatments, the steady-state P_700_^+^ level immediately before each SP was recorded.

### 4.4. Electrochromic Shift (ECS) Measurement

The ECS signal of carotenoid absorbance in the leaves attached to the plant was measured using the P515/535 modules of the DUAL-PAM 100 system. Plants were dark-adapted for 30 min, after which a single turnover flash was applied to determine the maximal ECS amplitude (ECS_ST_). Leaves were then illuminated at 163 µΕ m^−2^ s^−1^ for 5 or 10 min. The light was subsequently turned off for 60 s to record the ECS decay (representing pmf) and the subsequent partial recovery (representing the ΔpH component). All ECS signals were normalized to ECS_ST_ for the same leaf. The electric component (ΔΨ) was calculated as the difference between pmf and ΔpH.

### 4.5. Statistical Analysis

For FL experiments, ANOVA followed by the Holm–Bonferroni post hoc test was performed; different letters indicate significant differences (*p* < 0.05 or lower). For rSP and ECS measurements, genotype or treatment differences at each time point were assessed using a Two-Sample *t*-Test. Asterisks (*) indicate significant differences (*p* < 0.05 or lower); ns denotes not significant (*p* > 0.05).

## 5. Conclusions

Collectively, the results of this study, particularly the contrasting sensitivity of *tap38/pph1* relative to the WT under fluctuating light (FL) and repetitive short pulses (rSP), strongly demonstrate that FL and pulsed illumination constitute fundamentally distinct photoinhibitory stresses that engage different protective mechanisms. This finding underscores the necessity of clearly distinguishing between FL and rSP protocols to fully elucidate the spectrum of protective mechanisms that safeguard PSI under photoinhibitory conditions. Overall, the data support the conclusion that the protective strategy is determined by the site of primary photodamage: mechanisms regulating electron outflow from PSI are more crucial for protection during stresses that promote acceptor-side damage (e.g., FL), while mechanisms limiting electron supply to PSI are more crucial for protection during stresses that promote donor-side damage (e.g., rSP).

## Data Availability

The original contributions presented in this study are included in the article/Supplementary Materials. Further inquiries can be directed to the corresponding author.

## Supplementary Materials

The following supporting information can be downloaded at: https://www.mdpi.com/article/10.3390/ijms27010009/s1.

## Abbreviations

CBB cycle—Calvin–Benson–Bassham cycle; CET(I)—cyclic electron transfer around PSI; FL—fluctuating light; HL—high light; ISP—iron–sulfur protein; LHCII—light-harvesting complex II; LL—low light; PGR—Proton Gradient Regulation; PI(I)—photoinhibition of photosystem I; Pm—the maximal P700 oxidation level; PSI and PSII—photosystem I and photosystem II, respectively; rSP—repetitive short saturating pulse; SP—saturating pulse.
