# Supplementary material for: Donor- and Acceptor-Side Protection Against Photosystem I Photoinhibition in Arabidopsis thaliana
Source: Int J Mol Sci. 2025 Dec 19;27(1):9. doi: 10.3390/ijms27010009 (PMC12786166; doi:10.3390/ijms27010009)
Supplement: Supplementary file 1 [file ijms-27-00009-s001.zip › ijms-3984831-supplementary.pdf]

Donor- and Acceptor-Side Protection Against Photosystem I Photoinhibition in *Arabidopsis thaliana*

Marina Kozuleva

Institute of Basic Biological Problems of the Russian Academy of Sciences, Federal Research Center "Pushchino Scientific Center for Biological Research of the Russian Academy of Sciences", Pushchino, Russia

Supplementary Material

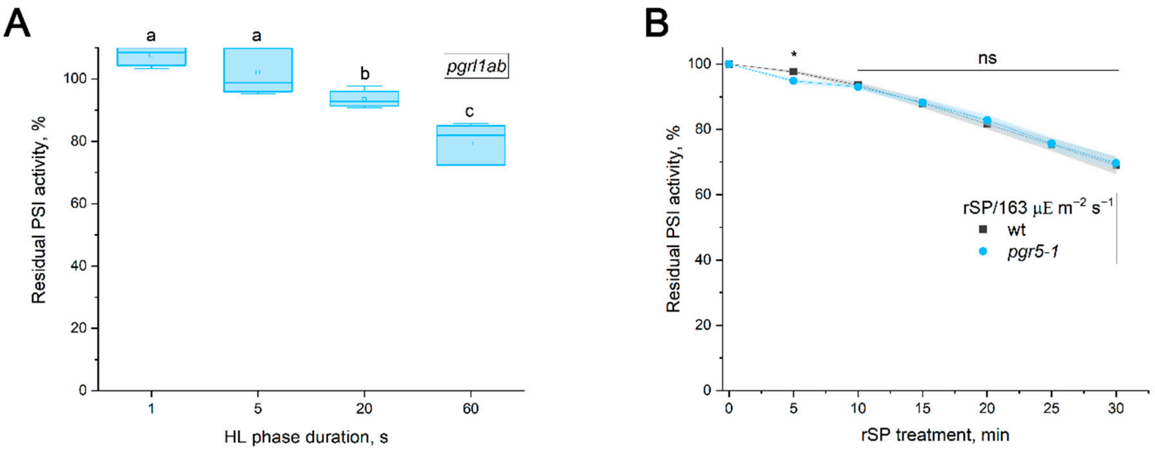

Supplementary Figure S1. (A) Dependence of FL-induced PI(I) on the HL phase duration in *pgrl1ab*. Boxplots show the median, mean, and interquartile range. Statistical significance was determined by ANOVA. Different letters indicate significant difference (p<0.05). (B) The effect of rSP applied at 163 µE m^{-2} s^{-1} on PSI activity in wild type (grey) and *pgr5-1* (blue). Data represent the mean of 5 individual leaves ± SE. Significant differences between each genotype at a given time point were determined by a Two Sample t-Test. Asterix (*) indicates significant difference with p<0.05, and ns indicates not significant (p>0.05).

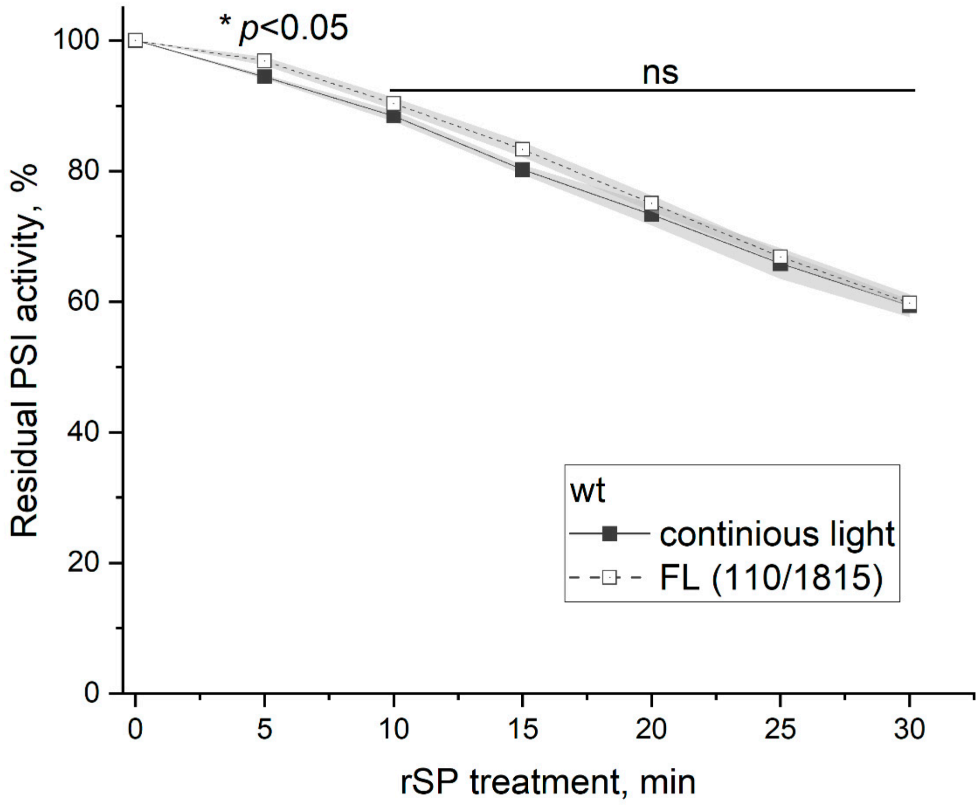

Supplementary Figure S2.

Effects of FL (four cycles of 110 μE m^{-2} s^{-1} light for 4 min and 1815 μE m^{-2} s^{-1} of light for 1 min) treatment on rSP-induced PI(I) in *Arabidopsis thaliana* leaves of WT.

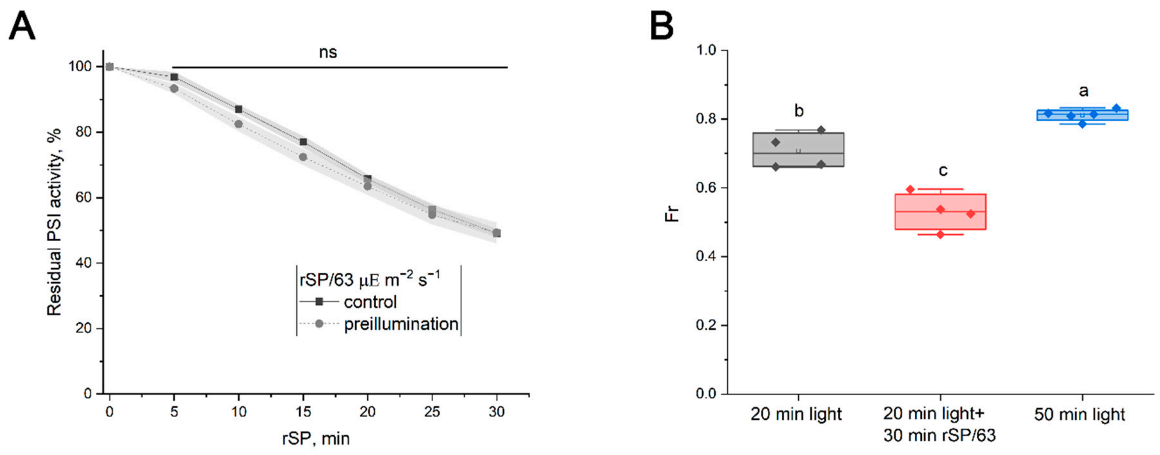

Supplementary Figure S3.
(A) - Effect of preillumination for 20 minutes with light at 63 μE m^{-2} s^{-1} on rSP-induced PSI activity loss in WT leaves.

Data represent means of 4 individual leaves ± SE. Significant genotype differences at each time point were determined by a Two Sample t-Test. ns indicates not significant (p>0.05).
(B) – Effect of light duration and rSP treatment on State 2 formation assessed as Fr parameter. Data represent individual values for 4-5 leaves per variant; boxplots show the median, mean, and interquartile range. Statistical significance was determined by ANOVA. Different letters indicate significant difference (p<0.05).

Fr parameter reflected changes in cChlorophyll *a* fluorescence of leaves induced by state transitions was measured as described earlier (Haldrup et al. 2001) with modification (Vetoshkina et al., 2024) using DUAL-PAM 100 (Walz). Briefly, dark-adapted leaves were exposed to light at 63 µE m^{-2} s^{-1} for 20 minutes, 20 minutes followed rSP/63 for 30 minutes, or 50 minutes. Then, the far-red light was switched on for 25 min followed the actinic light for 5 minutes.

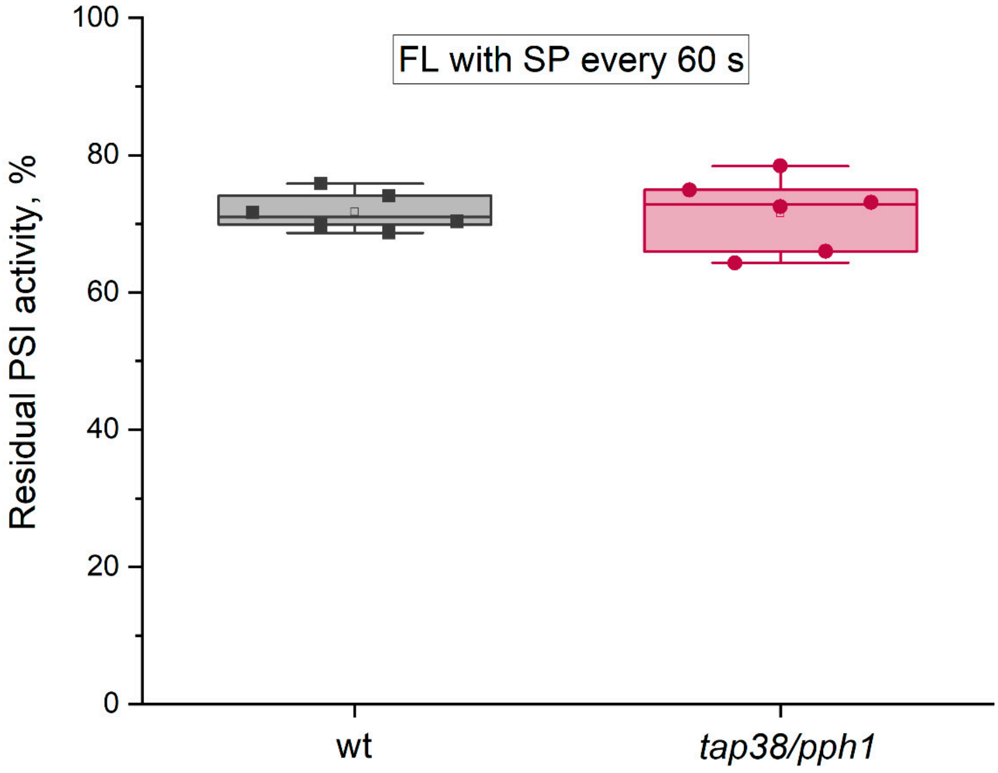

Supplementary Figure S4. The loss of PSI activity in *Arabidopsis thaliana* leaves of WT (grey squares) and *tap38/pph1* (red circles) induced with 7 cycles of FL with SP applied every 60 seconds. Data represent individual values for 4-5 leaves per variant; boxplots show the median, mean, and interquartile range.
